# Lightweight Semantic Perception from UAV-Borne Visual Sensors via Conflict-Suppressed Heterogeneous Expert Distillation

**DOI:** 10.3390/s26144509

**Published:** 2026-07-16

**Authors:** Feng Ouyang, Yongpeng Ding, Miao Qin, Weiting Xie, Chao Zhou

**Affiliations:** 1 College of Intelligent Technology, Tianfu College of Southwestern University of Finance and Economics, Mianyang 621000, China; ouyangfeng@tfswufe.edu.cn (F.O.); dingyongpeng@tfswufe.edu.cn (Y.D.); qinmiao@tfswufe.edu.cn (M.Q.); xieweiting@tfswufe.edu.cn (W.X.); 2 Mianyang Science and Technology City School-Enterprise Joint Application Technology Innovation Base for Low-Altitude Economy, Tianfu College of Southwestern University of Finance and Economics, Mianyang 621000, China; 3 School of Avionics and Electrical Engineering, Civil Aviation Flight University of China, Chengdu 610299, China

**Keywords:** UAV sensors, semantic segmentation, aerial perception, lightweight CNN, expert distillation, conflict suppression, edge deployment

## Abstract

UAV-borne visual sensors provide high-resolution aerial observations for low-altitude scene understanding, urban monitoring, traffic observation, emergency inspection, and infrastructure assessment. However, semantic perception from UAV visual sensor data remains challenging because aerial images often contain dense small objects, elongated road structures, fragmented boundaries, scale variations caused by flight-altitude changes, oblique viewpoints, and strict onboard or edge computational constraints. To address these challenges, this paper proposes MEKD-UAVSeg, a lightweight semantic perception framework based on conflict-suppressed heterogeneous expert distillation. During training, a Transformer-based semantic expert provides global contextual understanding and region-level class consistency, while a Mamba-based spatial expert provides complementary structural guidance for roads, roofs, boundaries, and other continuous aerial structures. Both experts are used only during training, and the final inference model remains a compact CNN-based segmentation network. In addition, UAV-aware density and hard-region priors are designed to emphasize small-object-dense areas, boundary-sensitive regions, rare classes, and uncertain aerial categories. A conflict-suppressed reliability routing strategy is further developed to reduce inconsistent supervision between heterogeneous experts and selectively transfer reliable knowledge to the student model. Experiments on UAVid and UDD6 demonstrate that the proposed framework achieves a favorable accuracy–efficiency trade-off compared with representative CNN-, Transformer-, Mamba-, and hybrid-based UAV segmentation methods, without introducing expert-induced inference complexity.

## 1. Introduction

UAV-borne visual sensors have become an important sensing modality for low-altitude environmental monitoring, traffic observation, emergency inspection, infrastructure assessment, and urban scene understanding. In these applications, aerial images captured by onboard cameras provide high-resolution visual sensor data for pixel-level semantic perception, which supports downstream tasks such as target-aware monitoring, road-region interpretation, obstacle-aware scene analysis, and mission-oriented decision assistance. Compared with ground-based visual sensors, UAV visual sensors acquire data from varying flight altitudes, oblique viewpoints, and wide-area perspectives, leading to large variations in object scale, dense small objects, elongated structures, and fragmented semantic boundaries [1,2,3]. Vehicles and pedestrians may occupy only a few pixels, whereas roads, roofs, rivers, and building contours often span long spatial ranges with weak or ambiguous boundaries. These characteristics make semantic perception from UAV visual sensor data particularly challenging, especially when the perception model must remain lightweight for resource-constrained onboard or edge sensing platforms.

Existing segmentation architectures face different limitations for UAV-borne visual sensing. Lightweight CNNs are efficient and easy to deploy on edge devices, but their local inductive bias and repeated downsampling often cause missed small targets, discontinuous road regions, and incomplete object boundaries [4,5,6]. Context-enhanced and multi-scale designs improve spatial coverage, but their aggregation patterns are still largely determined by fixed convolutional operations [7,8]. Transformer-based models provide stronger global semantic reasoning through token interaction [9,10,11]; however, their computational and memory costs are substantial for high-resolution aerial visual sensor data, and patch-level aggregation may weaken fine boundaries and small-object details. Recent state-space vision models show that selective scan can model long-range dependencies with favorable sequence complexity [12,13,14]. This property is useful for UAV sensing scenarios that contain long and direction-sensitive structures. Directly adding such modules to the inference network, however, increases deployment complexity. Therefore, a key question for UAV-borne intelligent sensing is how to exploit high-capacity semantic and spatial modeling during training while keeping the final visual perception model lightweight at inference time.

Knowledge distillation offers a practical way to improve a compact student without increasing inference cost. A common strategy is to train a lightweight model under the supervision of a stronger teacher and retain only the student during deployment [15,16,17]. For UAV semantic segmentation, however, single-teacher distillation has clear limitations. A larger Transformer teacher may provide stronger global semantics, but its knowledge is often entangled and does not explicitly distinguish region-level semantic consistency from direction-sensitive spatial continuity. A larger CNN teacher, even with stronger context modules, remains dominated by convolutional sampling and may provide limited guidance for long structured regions. Simply increasing teacher capacity, therefore, does not directly address the joint challenges of scale variation, small-object ambiguity, and structural fragmentation in UAV imagery.

Multi-teacher and multi-expert distillation have been explored to provide richer supervision for compact segmentation models [18,19]. Recent heterogeneous distillation methods further improve knowledge transfer across different architectures [20,21]. These methods, however, mainly focus on general teacher ensembles or cross-architecture feature alignment, rather than the UAV-specific conflict among semantic consistency, spatial continuity, and hard-region reliability. In UAV images, expert disagreement is strongly spatially dependent and often occurs around small objects, rare categories, thin structures, and ambiguous boundaries. Boundary- and hard-region-oriented distillation methods mitigate this issue by emphasizing difficult pixels or separating boundary and body regions [22,23]. In most cases, however, these cues are used only as additional supervision or loss reweighting. They are seldom integrated with expert feature construction and pixel-wise reliability routing. As a result, unreliable expert responses may still be transferred to the student in regions where UAV segmentation is most difficult.

The objective of this study is to improve the accuracy of lightweight UAV semantic perception without increasing inference-stage model complexity. The central research problem is how to transfer complementary semantic and spatial knowledge from high-capacity training-only experts to a compact deployable student, while suppressing unreliable supervision in small-object, boundary-rich, rare-class, and ambiguous UAV regions. This problem is different from simply designing a stronger segmentation backbone because the final model must remain efficient for UAV-borne visual sensing after all auxiliary expert branches are removed.

To address these issues, we propose MEKD-UAVSeg, a conflict-suppressed heterogeneous expert distillation framework for lightweight semantic perception from UAV-borne visual sensor data. Instead of relying on a single larger teacher or a homogeneous expert ensemble, the proposed framework decomposes teacher guidance into two role-specialized experts that are used only during training. The Transformer semantic expert focuses on region-level semantic consistency, while the Mamba spatial expert is used as a complementary training-only branch to provide additional spatial guidance for elongated and structurally continuous regions. We do not claim that Mamba is universally superior to all possible spatial modeling modules. Instead, it is adopted because its selective scanning introduces a spatial modeling bias different from both convolutional fixed-rate aggregation and Transformer token interaction. Since the Mamba branch is used only during offline training and removed after distillation, its additional cost does not affect the deployed visual sensing model. We further examine the necessity of this design through expert-configuration ablations, including single-expert, homogeneous dual-expert, heterogeneous expert settings, and alternative spatial expert designs.

The proposed framework also makes expert guidance UAV-aware and conflict-suppressed. To handle scale variation, expert-to-student knowledge is transferred across multiple feature levels. To focus supervision on failure-prone aerial regions, we construct UAV-aware structure and hard-region priors that jointly consider small-object density, boundary complexity, rare-class distribution, and prediction uncertainty. Unlike conventional hard-pixel reweighting, these priors are used not only to emphasize difficult pixels, but also to guide spatial expert feature fusion and reliability-aware knowledge transfer. A spatially adaptive conflict-suppressed distillation strategy then routes heterogeneous expert knowledge according to pixel-wise consistency and confidence, reducing unreliable supervision when experts disagree or when both experts are uncertain.

Overall, the central insight of this work is not to attach increasingly complex modules to the deployed visual sensing network, but to use heterogeneous high-capacity experts as training-only sources of complementary knowledge. From the perspective of UAV-borne visual sensor data processing, the proposed framework treats semantic segmentation as an edge-deployable aerial scene perception problem: semantic and spatial experts are separated according to their modeling biases, UAV-aware priors identify regions where expert reliability is likely to vary, and pixel-wise routing suppresses unreliable supervision before knowledge is transferred to the compact student. This formulation provides a practical route for improving semantic perception from UAV visual sensor data without increasing inference-stage parameters, FLOPs, or latency.

Our contributions are summarized as follows:

- We formulate UAV aerial semantic segmentation as a lightweight semantic perception problem for UAV-borne visual sensor data under onboard and edge computational constraints. Instead of increasing inference-stage model complexity, the proposed framework improves a compact CNN-based visual sensing model through training-only heterogeneous expert guidance.
- We design a role-specialized heterogeneous expert scheme for aerial visual sensor data processing, where a Transformer semantic expert provides global contextual understanding and region-level class consistency, while a Mamba spatial expert provides direction-sensitive structural guidance for elongated and continuous aerial patterns. Both experts are used only during training and are fully removed during deployment.
- We introduce UAV-aware structure and hard-region priors to describe small-object density, boundary complexity, rare-class distribution, and prediction uncertainty in aerial visual sensor data. Different from conventional hard-pixel reweighting, these priors are coupled with spatial expert feature fusion and pixel-wise reliability-aware knowledge transfer.
- We develop a conflict-suppressed distillation mechanism that routes heterogeneous expert knowledge according to expert consistency and confidence. Multi-scale feature distillation, logit distillation, boundary-aware supervision, and reliability routing are jointly used to reduce negative transfer in ambiguous UAV sensing regions.
- Extensive experiments on UAVid and UDD6 demonstrate that the proposed method achieves a favorable accuracy–efficiency trade-off for UAV-borne visual sensing. The final inference model achieves 69.17% mIoU on UAVid and 80.68% mIoU on UDD6 with only 16.45 M parameters, indicating its potential for edge-deployable aerial scene understanding.

## 2. Related Work

### 2.1. UAV Semantic Segmentation

Semantic segmentation of UAV imagery has been widely studied for low-altitude perception. UAVid provides high-resolution oblique-view urban UAV scenes with dense pixel-level annotations and has become a representative benchmark for UAV semantic segmentation [1]. Recent reviews summarize UAV semantic segmentation methods and datasets and point out that small-object recognition, boundary preservation, efficiency, and cross-scene robustness remain open challenges [2]. Compared with satellite or ground-view imagery, UAV imagery contains stronger viewpoint changes, larger scale variations, and more fragmented object layouts, which require both local detail preservation and long-range context modeling.

In addition to UAVid, several drone-view datasets have been introduced to broaden the evaluation scenarios of UAV semantic segmentation. UDD6 provides UAV-captured urban scenes with six semantic categories and is commonly used to evaluate segmentation models under low-altitude viewpoints. VDD further extends drone semantic segmentation to more varied scenes, camera angles, illumination conditions, and weather conditions, and provides high-resolution pixel-level annotations for multiple land-cover and object categories [24]. These datasets indicate that UAV semantic segmentation models should not only perform well on a single urban benchmark but also remain robust to cross-scene variations, class imbalance, and scale changes.

CNN-based methods remain attractive for UAV and remote sensing segmentation because of their efficiency and deployment friendliness. Real-time segmentation models such as BiSeNetV2, STDC, DDRNet, and PIDNet improve the accuracy–efficiency trade-off through bilateral paths, lightweight feature aggregation, or boundary-aware branches [4,5,6,25]. For remote sensing imagery, ABCNet and UNetFormer further introduce efficient context modeling and global–local aggregation to handle fine-resolution scenes [7,8]. EMNet improves UAV remote sensing segmentation by fusing edge features and multi-level upsampling [26]. These methods improve compact segmentation networks, but purely convolutional designs may still miss long-range structural continuity in aerial scenes.

Transformer-based segmentation models have also been applied to dense prediction. Swin Transformer, Segmenter, SegFormer, Mask2Former, and TopFormer show that token interaction can improve global semantic aggregation and dense prediction accuracy [9,10,11,27,28]. RTFormer and SeaFormer further study efficient Transformer designs for real-time or mobile semantic segmentation [29,30]. In UAV segmentation, PPTFormer constructs pseudo multi-perspective representations to address viewpoint changes in UAV-captured scenes [3]. UAV-FAENet introduces frequency-aware and attention-enhanced modules to improve small-target recognition and boundary quality [31]. Mamba-UAV-SegNet explores state-space modeling for real-time UAV aerial image segmentation [32]. These methods demonstrate the value of stronger context modeling, but most of them improve accuracy by modifying the inference network itself. In contrast, MEKD-UAVSeg improves a lightweight CNN student through training-only expert distillation, and the expert branches are removed after training.

Recent studies on UAV and remote sensing visual understanding further show the importance of long-range contextual modeling and boundary-aware structural representation. Li et al. [33] investigated unpaved road segmentation from UAV imagery using a global vision Transformer with dilated cross-window self-attention, demonstrating the effectiveness of global context modeling for aerial road perception and dynamic mapping. Zhao et al. [34] proposed BGFNet for remote sensing semantic segmentation by incorporating boundary information and graph structure fusion, highlighting the role of boundary-aware structural cues in high-resolution aerial scenes. These studies are closely related to the challenges addressed in this work, including long-range structure modeling, boundary preservation, and aerial scene parsing. Different from these inference-time architecture designs, our framework improves a deployable CNN student through training-only heterogeneous expert distillation, where Transformer and Mamba experts provide complementary semantic and spatial guidance without increasing inference-stage complexity.

### 2.2. Knowledge Distillation for Compact Segmentation

Knowledge distillation is commonly used to improve compact models by transferring knowledge from stronger teachers. For dense prediction, distillation is more difficult than classification because the student must learn both semantic discrimination and spatially aligned structural cues. Channel-wise distillation transfers category-aware activation patterns through channel-wise probability distributions [15]. CIRKD transfers cross-image pixel-to-pixel and pixel-to-region relations to improve the semantic structure of the student feature space [16]. IFVD transfers intra-class feature variation, while MGD reconstructs masked student features under teacher supervision [35,36]. DKD further decouples logit distillation into target-class and non-target-class components [17]. These methods provide useful tools for compact segmentation, but most of them still follow a single-teacher setting.

Several works focus on structural or hard-region distillation. BPKD separates boundary and body regions and distills boundary-privileged knowledge to improve spatially sensitive areas [22]. BRD combines boundary distillation and relation distillation to address boundary incompleteness and region connectivity errors in compact segmentation models [23]. FAKD enhances teacher–student feature transfer by augmenting feature representations for semantic segmentation [37]. These methods show that treating all pixels equally is suboptimal. However, boundary and hard-region cues are usually used as additional supervision or loss reweighting masks. They are rarely used to construct expert features or to estimate expert reliability during knowledge transfer.

Multi-teacher and heterogeneous distillation methods aim to provide richer supervision. MTED transfers output-level and feature-level knowledge from multiple teachers to a compact segmentation student [18]. MTKD-RL formulates teacher weighting as a reinforcement learning problem and dynamically balances teacher contributions [19]. HeteroAKD studies semantic segmentation distillation from heterogeneous architectures, and PAT adapts teacher features to students with different architectural perspectives [20,21]. These methods show the importance of teacher selection and cross-architecture adaptation. However, most of them are designed for generic heterogeneous transfer and do not explicitly model the spatially varying reliability of experts in UAV imagery. Although existing distillation methods have improved compact segmentation models, they usually transfer knowledge from a single teacher or combine multiple teachers with global weighting strategies. Such designs do not explicitly consider that teacher reliability may vary spatially across UAV scenes. In particular, small objects, rare categories, thin boundaries, elongated roads, and roof–road transition regions may cause different experts to produce inconsistent or uncertain responses. Boundary-aware distillation methods improve spatially sensitive regions, but boundary cues are commonly used as additional loss terms or reweighting masks rather than as factors for expert construction and reliability routing. In contrast, the proposed framework couples UAV-aware priors with heterogeneous expert fusion and pixel-wise conflict suppression, allowing the student to receive reliable semantic and spatial knowledge selectively instead of indiscriminately absorbing all teacher responses.

### 2.3. Visual State Space Models for Dense Prediction

State space models have recently become an efficient alternative to attention-based long-sequence modeling. Mamba introduces input-dependent selective state-space modeling and achieves linear-time sequence modeling [12]. Vision Mamba and VMamba extend Mamba-style modeling to visual representation learning through bidirectional or two-dimensional selective scanning [13,14]. Compared with CNNs based on fixed local sampling and Transformers based on token interaction, visual state space models provide a different spatial modeling bias for long-range dependency propagation.

The long-range modeling ability and linear complexity of state space models are particularly useful for high-resolution remote sensing dense prediction. RS-Mamba designs remote sensing Mamba modules for large, very-high-resolution remote sensing images [38]. RS3Mamba introduces a visual state-space branch into remote sensing semantic segmentation to provide global information for a convolutional main branch [39]. Samba and UNetMamba further explore Mamba-based encoder–decoder structures for high-resolution remote sensing semantic segmentation [40,41]. These methods suggest that Mamba-style models can capture global context and structured spatial dependencies in dense prediction tasks.

Despite these advances, existing visual and remote sensing Mamba methods usually use Mamba as part of the inference network. This strategy improves representation ability but changes the deployed architecture and may introduce additional inference cost. For UAV deployment, where computation, memory, and latency are important constraints, directly adding Mamba modules to the inference branch is not always desirable. Our work takes a different route. We use Mamba as a training-only spatial expert rather than an inference-time backbone component. The Mamba branch provides direction-sensitive structural supervision for roads, roofs, boundaries, and other continuous aerial patterns during offline optimization and is discarded after distillation. Therefore, the deployed model remains a compact CNN student while benefiting from state-space structural guidance.

## 3. Method

### 3.1. Framework Overview

**Figure 1:**
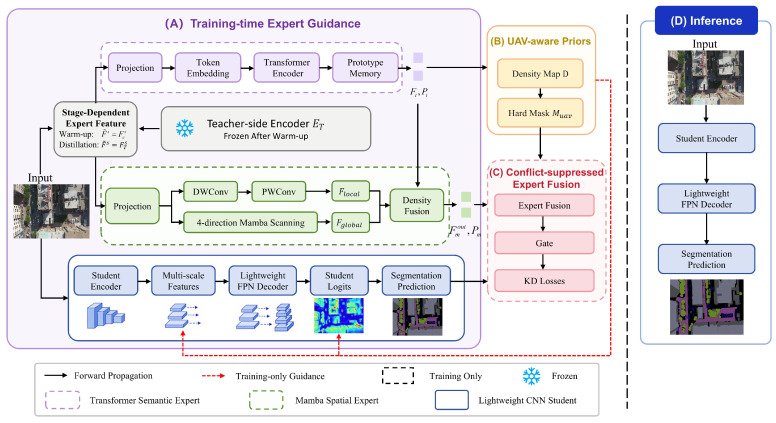
Overall architecture of MEKD-UAVSeg. During training, Transformer and Mamba experts provide complementary semantic and spatial guidance to a lightweight CNN student through UAV-aware priors and conflict-suppressed expert fusion. During inference, all expert branches, prior modules, routing modules, and distillation losses are discarded, leaving only the lightweight CNN student for semantic perception.

MEKD-UAVSeg is a lightweight semantic perception framework for UAV-borne visual sensor data. Given an input image $I\in R^{3\times H\times W}$ captured by an onboard UAV camera and its label map $Y\in {\left\{1,\ldots ,K\right\}}^{H\times W}$, the goal is to predict a dense semantic map $\hat{Y}$ for aerial scene understanding. The framework includes a lightweight convolutional neural network (CNN) student and two heterogeneous experts used only during training: a Transformer semantic expert and a Mamba spatial expert. At inference time, only the student branch is retained. The two experts are discarded after training and serve only to provide complementary semantic and spatial supervision. Figure 1 illustrates the overall architecture of the proposed framework, including training-time expert guidance, UAV-aware prior construction, conflict-suppressed expert fusion, and deployment-time CNN-only inference.

The term heterogeneity-aware refers to the use of two experts with different modeling biases rather than two interchangeable network blocks. The Transformer expert models global pairwise token interactions and uses class prototypes to improve region-level semantic consistency. The Mamba expert performs selective state-space modeling along spatial sequences and uses structure-density priors to better capture direction-sensitive spatial patterns. The term conflict-suppressed means that the two expert predictions are not simply averaged. Instead, unreliable or inconsistent expert responses are assigned lower weights through reliability-aware routing and adaptive distillation gating.

To separate the warm-up and distillation stages, we use a stage-dependent expert input. During warm-up, the experts receive features from the current student encoder. After warm-up, the student encoder is copied to initialize a teacher-side encoder, which is then frozen:

(1) $$E_{T}\leftarrow E_{c}.$$

The expert input feature at scale *s* is defined as

(2) $${\tilde{F}}^{s}=\left\{\begin{array}{cc} F_{c}^{s}=E_{c}^{s}\left(I\right), & \mathrm{warm-up},\\ F_{T}^{s}=E_{T}^{s}\left(I\right), & \mathrm{distillation}. \end{array}\right.$$

This design avoids using an uninitialized teacher encoder during warm-up and provides stable expert inputs during distillation. Unless otherwise specified, features, logits, and prior maps used in element-wise operations or concatenation are resized to a common spatial resolution by the resize operator R(·;·), where the second argument denotes the reference feature map. The teacher-side encoder $E_{T}$ and all expert modules are removed after training. During inference, the prediction is produced only by the CNN student:

(3) $$\hat{Y}={\mathrm{Dec}}_{c}\left(E_{c}\left(I\right)\right).$$

### 3.2. Lightweight Student Segmenter

The CNN student is the final deployable segmentation model. It can be instantiated with efficient CNN backbones such as MobileNetV3 [42], STDCNet [4], EfficientNet-Lite [43], or ResNet18 [44]. In this work, STDC2-FPN is selected as the default student because STDCNet provides a strong accuracy–efficiency trade-off in real-time semantic segmentation, and the lightweight FPN decoder enables multi-scale feature fusion with limited additional inference cost. Given *I*, the student encoder extracts multi-scale features:

(4) $$\left\{F_{c}^{1/4},F_{c}^{1/8},F_{c}^{1/16},F_{c}^{1/32}\right\}=E_{c}\left(I\right),$$

where the superscript denotes the spatial resolution relative to the input image. Low-level features retain local textures, object boundaries, and small-object details, while high-level features provide stronger semantic context.

The decoder follows a lightweight feature pyramid design with 1×1 projections, depthwise separable convolutions, bilinear upsampling, and skip connections. No Transformer, Mamba, or heavy attention module is added to the inference branch. The student logits are computed as

(5) $$P_{c}={\mathrm{Dec}}_{c}\left(F_{c}^{1/4},F_{c}^{1/8},F_{c}^{1/16},F_{c}^{1/32}\right).$$

Since UAV imagery often has severe class imbalance, the student is trained with a class-balanced segmentation loss:

(6) $$L_{seg}^{c}=L_{cb-ce}\left(P_{c},Y\right)+\lambda_{dice}L_{dice}\left(P_{c},Y\right).$$

The class-balanced weight for class *k* is defined as

(7) $$\omega_{k}=\frac{1}{\log(\mu +f_{k})},$$

where $f_{k}$ denotes the frequency of class *k* in the training set, μ is a smoothing constant, and the corresponding pixel-wise weight is denoted by $\omega_{Y_{i}}$.

### 3.3. Role-Specialized Heterogeneous Experts

The Transformer semantic expert is used to improve region-level semantic consistency. UAV scenes often contain visually confusing regions, such as roofs and roads, vegetation and bare land, or small vehicles and background clutter. To aggregate semantic context, the Transformer expert takes high-level stage-dependent features as input:

(8) $$F_{t}^{in}=\phi_{t}\left([{\tilde{F}}^{1/16},\mathrm{Up}\left({\tilde{F}}^{1/32}\right)]\right),$$

where $\phi_{t}\left(\cdot \right)$ is a lightweight projection layer and Up(·) denotes bilinear upsampling. The projected feature is flattened into tokens, encoded by a lightweight Transformer encoder, and reshaped into a two-dimensional feature map:

(9) $$F_{t}=\mathrm{Reshape}\left(\mathrm{TransEnc}(\mathrm{Flatten}\left(F_{t}^{in}\right)+E_{pos})\right),\;P_{t}={\mathrm{Head}}_{t}\left(F_{t}\right).$$

Prototype learning is introduced only after the Transformer expert has been warmed up, since small and rare UAV classes may produce unstable prototypes at the beginning of training. During the initial warm-up epochs, the Transformer expert is supervised only by the class-balanced segmentation loss. After this preliminary warm-up, the prototype memory is initialized with Transformer expert features from the training set:

(10) $$p_{k}^{0}=\frac{\sum_{\left(I,Y\right)\in D_{tr}}\sum_{i}⊮\left(Y_{i}=k\right)F_{t}^{i}}{\sum_{\left(I,Y\right)\in D_{tr}}\sum_{i}⊮\left(Y_{i}=k\right)+\epsilon }.$$

In the following epochs, the batch-wise prototype ${\hat{p}}_{k}$ updates the memory only when the number of valid pixels $N_{k}$ is larger than $n_{min}$:

(11) $$p_{k}\leftarrow \eta p_{k}+\left(1-\eta \right){\hat{p}}_{k}.$$

If $N_{k}\leq n_{min}$, the stored prototype remains unchanged. The class-balanced prototype objective is

(12) $$L_{proto}=-\sum_{i}\omega_{Y_{i}}\log\frac{\exp(\mathrm{sim}\left(F_{t}^{i},p_{Y_{i}}\right)/\tau )}{\sum_{k=1}^{K}\exp\left(\mathrm{sim}\left(F_{t}^{i},p_{k}\right)/\tau \right)}.$$

The Transformer expert is supervised by

(13) $$L_{seg}^{t}=L_{cb-ce}\left(P_{t},Y\right)+⊮\left(e\geq e_{\mathrm{proto}}\right)\lambda_{proto}L_{proto},$$

where *e* denotes the current epoch and $e_{\mathrm{proto}}$ denotes the epoch after which the initialized prototype memory becomes available.

The Mamba spatial expert is used as a training-only spatial modeling branch to provide complementary structural guidance for elongated UAV patterns. Rather than treating Mamba as an inherently superior backbone, we use it to introduce an input-dependent selective-scan bias that differs from convolutional and attention-based aggregation. It receives the same stage-dependent high-level features:

(14) $$F_{m}^{in}=\phi_{m}\left([{\tilde{F}}^{1/16},\mathrm{Up}\left({\tilde{F}}^{1/32}\right)]\right).$$

The local path uses depthwise separable convolution to extract fine-grained structures:

(15) $$F_{local}=\mathrm{PWConv}\left({\mathrm{DWConv}}_{3\times 3}\left(F_{m}^{in}\right)\right).$$

The global path applies Mamba scanning along four directions. Direction-adaptive weights are predicted from $F_{m}^{in}$ and used to aggregate directional responses:

(16) $$F_{global}=\sum_{r\in \{lr,rl,tb,bt\}}\omega_{r}F_{r},\;\left\{\omega_{r}\right\}=\mathrm{Softmax}\left(\psi_{dir}\left(F_{m}^{in}\right)\right).$$

This design helps the Mamba expert represent elongated structures such as roads, rivers, field boundaries, and building contours.

### 3.4. UAV-Aware Structure and Hard-Region Priors

Before presenting the formal definitions, we first give an intuitive explanation of the UAV-aware priors. UAV segmentation errors often concentrate in four types of regions: dense small objects, fragmented boundaries, rare categories, and uncertain predictions. These regions are difficult not only because they are visually ambiguous, but also because different experts may provide inconsistent supervision. Therefore, the proposed priors are not used merely to increase the loss weight of hard pixels. Instead, they serve two functions. First, they guide the Mamba spatial expert to allocate more structural modeling capacity to boundary-rich and small-object-dense regions. Second, they are fed into the expert router so that knowledge transfer becomes spatially adaptive. In this way, the framework emphasizes difficult UAV regions while suppressing unreliable expert responses in pixels where semantic and spatial experts disagree.

To guide the spatial expert, we build a structure-density map that highlights boundary-rich, small-object-dense, and locally complex regions. The predicted density map is generated from the stage-dependent low-level expert feature:

(17) $$D=\sigma (\psi_{d}\left({\tilde{F}}^{1/8}\right)).$$

The pseudo target $D^{*}$ is constructed from three label-derived cues: boundary density B(Y), small-object density S(Y), and local semantic complexity C(Y). The boundary cue is obtained with fixed Sobel kernels:

(18) $$\mathrm{Edge}\left(P\right)=\sqrt{{\left(S_{x}*P\right)}^{2}+{\left(S_{y}*P\right)}^{2}+\epsilon },\;B\left(Y\right)=\mathrm{Dilate}\left(\mathrm{Edge}\left(Y^{oh}\right)\right),$$

where $S_{x}$ and $S_{y}$ are non-learnable Sobel kernels and $Y^{oh}$ is the one-hot label map. The Sobel operator, connected-component analysis, and entropy-based local complexity are not claimed as new operators in this work. They are used as simple and efficient label-derived cues to construct UAV-aware supervision priors. The novelty lies in coupling these priors with spatial expert feature fusion and reliability-aware knowledge transfer, rather than using them only as standalone boundary losses or hard-pixel reweighting terms. When Sobel filtering is applied to one-hot labels, it is used only to generate a non-gradient pseudo-target for density supervision. When it is applied to predicted probability maps in boundary-aware distillation, the operation is differentiable with respect to the predicted probabilities.

For small-object density, connected components $\Omega_{n}$ are extracted from *Y*, and the small-object threshold is defined by the area quantile of connected components in the training set:

(19) $$\tau_{s}=Q_{q}\left(\left\{\mathrm{Area}\left(\Omega_{n}\right)\right\}\right),\;S_{i}\left(Y\right)=\sum_{n}⊮\left(\mathrm{Area}\left(\Omega_{n}\right)<\tau_{s}\right)⊮\left(i\in \Omega_{n}\right).$$

The local semantic complexity C(Y) is computed by multi-window local class entropy. To reduce the influence of overlapping cues, the three cues are first normalized to $\tilde{B}$, $\tilde{S}$, and $\tilde{C}$ by percentile clipping, and then combined through a soft-union operation:

(20) $$D^{*}={\mathrm{Norm}}_{q}\left(1-\left(1-\tilde{B}\right)\left(1-\tilde{S}\right)\left(1-\tilde{C}\right)\right),$$

where ${\mathrm{Norm}}_{q}\left(\cdot \right)$ denotes percentile-based normalization. This formulation avoids excessive accumulation in regions where boundary, small-object, and complexity cues overlap. The density supervision is defined as a balanced binary cross-entropy loss:

(21) $$L_{den}={\mathrm{BCE}}_{bal}\left(D,D^{*}\right).$$

Before being used in the Mamba expert, the density map is resized to the resolution of $F_{m}^{in}$:

(22) $$D_{m}=R\left(D;F_{m}^{in}\right).$$

The Mamba expert uses $D_{m}$ for residual density-guided fusion:

(23) $$F_{m}^{out}=F_{m}^{in}+\gamma_{l}D_{m}⊙F_{local}+\gamma_{g}\left(1-D_{m}\right)⊙F_{global}+\phi_{int}\left(\left[F_{local},F_{global},D_{m}\right]\right).$$

Here, $D_{m}$ is broadcast along the channel dimension in element-wise multiplication. The Mamba expert prediction is

(24) $$P_{m}={\mathrm{Head}}_{m}\left(F_{m}^{out}\right),\;L_{seg}^{m}=L_{cb-ce}\left(P_{m},Y\right)+\lambda_{den}L_{den}.$$

We further construct a UAV hard-region mask to assign closer attention to pixels that require more careful knowledge transfer. The mask combines small-object regions, boundary regions, rare classes, and uncertain regions:

(25) $$M_{uav}={\mathrm{Norm}}_{q}\left(1-\prod_{j\in \{s,b,r,h\}}\left(1-{\tilde{M}}_{j}\right)\right),$$

where ${\tilde{M}}_{s}$, ${\tilde{M}}_{b}$, ${\tilde{M}}_{r}$, and ${\tilde{M}}_{h}$ denote normalized small-object, boundary, rare-class, and uncertainty masks, respectively. Specifically, ${\tilde{M}}_{s}$ and ${\tilde{M}}_{b}$ are derived from S(Y) and B(Y), while ${\tilde{M}}_{r}$ is derived from the class-balanced weight $\omega_{Y_{i}}$. The raw uncertainty mask is computed from the student prediction:

(26) $$M_{h}=1-\max_{k}\mathrm{Softmax}{\left(P_{c}\right)}_{k}.$$

Because student predictions are unstable at the beginning of distillation, the uncertainty mask is disabled in the early distillation epochs and activated only after the distillation losses become relatively stable. This delayed activation prevents early noisy student predictions from dominating the hard-region prior. Before the activation epoch $e_{\mathrm{act}}$, we set ${\tilde{M}}_{h}=0$. After that epoch, the normalized uncertainty mask is computed as

(27) $${\tilde{M}}_{h}={\mathrm{Norm}}_{q}\left(M_{h}\right).$$

In the default setting, $e_{\mathrm{act}}$ is set to 10 epochs after the beginning of the distillation stage. A sensitivity analysis of this activation epoch is provided in Section 4.4.

**Figure 2:**
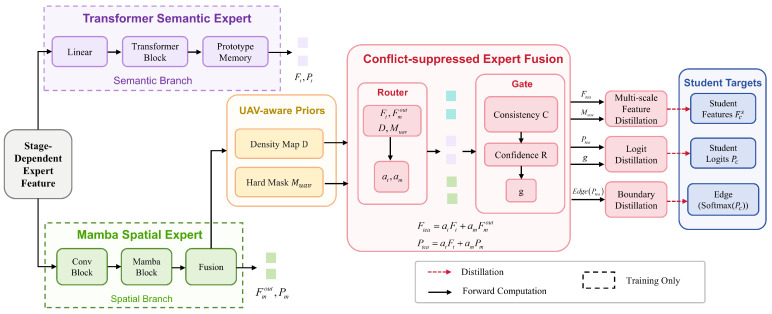
Architecture of the heterogeneous expert fusion and conflict-suppressed distillation module in MEKD-UAVSeg. The figure illustrates the Transformer semantic expert, Mamba spatial expert, UAV-aware priors, expert router, consistency–confidence gate, and student targets, where reliable expert cues are transferred to the student through logit, multi-scale feature, and boundary distillation.

Figure 2 further details the heterogeneous expert fusion and conflict-suppressed distillation module, where reliable expert cues are transferred to the student through logit, multi-scale feature, and boundary distillation.

### 3.5. Conflict-Suppressed Expert Distillation

The two experts may make complementary but sometimes conflicting predictions. To reduce negative transfer, we use a reliability-gated distillation mechanism. Before routing, the structure-density map and UAV hard-region mask are resized to the expert feature resolution:

(28) $$D_{e}=R\left(D;F_{t}\right),\;M_{uav}^{e}=R\left(M_{uav};F_{t}\right).$$

The expert router predicts spatially adaptive weights from the expert features, the structure-density map, and the UAV hard-region mask:

(29) $$\left[\alpha_{t},\alpha_{m}\right]=\mathrm{Softmax}\left(\mathrm{Router}(\left[F_{t},F_{m}^{out},D_{e},M_{uav}^{e}\right])\right).$$

The fused teacher feature is computed as

(30) $$F_{tea}=\alpha_{t}F_{t}+\alpha_{m}F_{m}^{out}.$$

For logit fusion, the routing weights are resized to the logit resolution:

(31) $$\left[\alpha_{t}^{p},\alpha_{m}^{p}\right]=R\left(\left[\alpha_{t},\alpha_{m}\right];P_{t}\right),\;P_{tea}=\alpha_{t}^{p}P_{t}+\alpha_{m}^{p}P_{m}.$$

Expert consistency is measured by the Jensen–Shannon divergence between the expert predictions:

(32) $$C=1-\mathrm{JS}\left(Q_{t},Q_{m}\right),\;Q_{t}=\mathrm{Softmax}\left(P_{t}\right),\;Q_{m}=\mathrm{Softmax}\left(P_{m}\right).$$

To avoid batch-wise oscillation, the consistency threshold is updated by an exponential moving average:

(33) $${\bar{\delta }}_{c}\leftarrow m{\bar{\delta }}_{c}+\left(1-m\right)Q_{0.1}\left(C\right).$$

The expert confidence is defined as

(34) $$R_{i}=\max\left\{\max_{k}Q_{t}^{i}\left(k\right),\max_{k}Q_{m}^{i}\left(k\right)\right\},$$

and its threshold ${\bar{\delta }}_{r}$ is updated in the same way. The reliability gate is defined as

(35) $$g_{i}=\sigma \left(\frac{C_{i}-{\bar{\delta }}_{c}}{\tau_{c}}\right)\cdot \sigma \left(\frac{R_{i}-{\bar{\delta }}_{r}}{\tau_{r}}\right),$$

which weakens distillation at pixels where the experts strongly disagree or where both experts are uncertain. The two temperatures $\tau_{c}$ and $\tau_{r}$ control the smoothness of the consistency gate and confidence gate, respectively. We set $\tau_{c}=\tau_{r}=0.2$ as the default value to avoid introducing extra hyperparameter asymmetry into the routing module. This setting does not assume that consistency and confidence have identical distributions; instead, the two quantities are first normalized by their own EMA-updated thresholds ${\bar{\delta }}_{c}$ and ${\bar{\delta }}_{r}$, and the shared temperature only controls the transition sharpness after threshold normalization. A sensitivity analysis of different temperature settings is reported in Section 4.4.

The class-balanced and region-weighted logit distillation loss is

(36) $$L_{logit}=T^{2}\sum_{i}\omega_{Y_{i}}g_{i}\left(1+\beta M_{uav}^{i}\right)\mathrm{KL}\left(\mathrm{Softmax}\left(\mathrm{sg}\left(P_{tea}^{i}\right)/T\right)\|\mathrm{Softmax}\left(P_{c}^{i}/T\right)\right).$$

For feature-level transfer, the student and teacher features are aligned at multiple scales through learnable projection layers. The multi-scale feature distillation loss is

(37) $$L_{msfd}=\sum_{s\in \{1/8,1/16,1/32\}}\lambda_{s}{∥\omega_{Y}^{s}M_{uav}^{s}⊙\left(\phi_{c}^{s}\left(F_{c}^{s}\right)-\mathrm{sg}\left(F_{tea}^{s}\right)\right)∥}_{2}^{2}.$$

Here, $F_{\mathrm{tea}}^{s}$ denotes the fused teacher feature aligned to scale *s* by scale-specific projection and resizing. The class-balanced weight $\omega_{Y}$ and the UAV hard-region mask $M_{\mathrm{uav}}$ are resized to the spatial resolution of scale *s* to obtain $\omega_{Y}^{s}$ and $M_{\mathrm{uav}}^{s}$. Nearest-neighbor interpolation is used for $\omega_{Y}$, and bilinear interpolation is used for the soft mask $M_{\mathrm{uav}}$. To preserve fine contours, we further use a boundary-aware distillation term:

(38) $$L_{bd}={∥\mathrm{Edge}\left(\mathrm{Softmax}\left(P_{c}\right)\right)-\mathrm{sg}\left(\mathrm{Edge}\left(\mathrm{Softmax}\left(P_{tea}\right)\right)\right)∥}_{1}.$$

The router is supervised by training-only reliability targets derived from expert errors. The ground-truth label is used only to train the routing module and is not used during inference. The pixel-wise expert errors are

(39) $$e_{t}^{i}=L_{ce}\left(P_{t}^{i},Y_{i}\right),\;e_{m}^{i}=L_{ce}\left(P_{m}^{i},Y_{i}\right),$$

and the soft reliability target is

(40) $$r_{t}^{i}=\frac{\exp(-e_{t}^{i}/\tau_{\alpha })}{\exp\left(-e_{t}^{i}/\tau_{\alpha }\right)+\exp\left(-e_{m}^{i}/\tau_{\alpha }\right)},\;r_{m}^{i}=1-r_{t}^{i}.$$

To prevent the routing objective from changing the expert predictions, the target is detached:

(41) $$L_{route}=\mathrm{KL}\left(\mathrm{sg}\left(\left[r_{t},r_{m}\right]\right)\|\left[\alpha_{t},\alpha_{m}\right]\right).$$

### 3.6. Training Protocol and Deployment

The training process has two stages: warm-up and conflict-suppressed distillation. In the warm-up stage, the expert input features are extracted from the current student encoder, namely ${\tilde{F}}^{s}=F_{c}^{s}$. The CNN student, Transformer semantic expert, and Mamba spatial expert are jointly optimized by

(42) $$L_{warm}=L_{seg}^{c}+\lambda_{t}L_{seg}^{t}+\lambda_{m}L_{seg}^{m}.$$

During the initial part of warm-up, the prototype term in $L_{seg}^{t}$ is disabled by $⊮(e\geq e_{\mathrm{proto}})=0$. After the preliminary warm-up, the prototype memory is initialized with Transformer expert features from the training set, and the prototype objective is activated. This avoids using uninitialized prototype memory and allows the Transformer expert to receive region-level semantic regularization in later training epochs. In this stage, both experts learn semantic and spatial knowledge under the same feature distribution as the student encoder.

After warm-up, the teacher-side encoder is initialized as $E_{T}\leftarrow E_{c}$. During the distillation stage, $E_{T}$ and the two experts are frozen, and the expert input features are produced by the teacher-side encoder, namely ${\tilde{F}}^{s}=F_{T}^{s}$. The student encoder, routing module, and distillation adapters are optimized by

(43) $$L_{dist}=L_{seg}^{c}+\lambda_{ms}L_{msfd}+\lambda_{logit}L_{logit}+\lambda_{bd}L_{bd}+\lambda_{r}L_{route}.$$

The distillation weights are gradually increased at the beginning of this stage to avoid imposing overly strong constraints from fixed teacher representations. This allows the student to use stable expert knowledge while keeping the flexibility of task-driven optimization.

During inference, the Transformer expert, Mamba expert, teacher-side encoder, structure-density predictor, expert router, auxiliary heads, and all distillation-related modules are discarded. The deployed model contains only the lightweight CNN encoder and decoder:

(44) $$\hat{Y}={\mathrm{Dec}}_{c}\left(E_{c}\left(I\right)\right).$$

MEKD-UAVSeg, therefore, improves the semantic perception accuracy of the lightweight student through offline heterogeneous expert distillation while maintaining the inference efficiency needed for UAV-borne visual sensing.

To make the overall training and deployment procedure easier to follow, Algorithm 1 summarizes the proposed conflict-suppressed heterogeneous expert distillation process. The algorithm highlights the warm-up stage, UAV-aware prior construction, heterogeneous expert guidance, conflict-suppressed routing, and deployment-time removal of all expert and distillation modules.
**Algorithm 1** Conflict-Suppressed Heterogeneous Expert Distillation.**Require:** Training image *I*, label map *Y*, CNN student *S*, Transformer semantic expert $E_{t}$, and Mamba spatial expert $E_{m}$ **Ensure:** Deployable lightweight CNN student *S*  1:**Stage 1: Warm-up training**  2:Train *S*, $E_{t}$, and $E_{m}$ using the class-balanced segmentation objective.  3:Feed the experts with features from the current student encoder.  4:Copy the student encoder to initialize the teacher-side encoder after warm-up.  5:Freeze the teacher-side encoder and the two expert branches.  6:**Stage 2: UAV-aware prior construction**  7:Construct boundary-density, small-object-density, rare-class, and uncertainty cues from labels and student predictions.  8:Fuse these cues into the structure-density map *D* and the UAV-aware hard-region prior $M_{uav}$.  9:Use *D* and $M_{uav}$ to identify regions where segmentation errors and expert conflicts are more likely to occur.10:**Stage 3: Heterogeneous expert guidance**11:Use the Transformer semantic expert $E_{t}$ to generate region-level semantic features and logits.12:Use the Mamba spatial expert $E_{m}$ to generate direction-sensitive structural features and logits under density-guided feature fusion.13:**Stage 4: Conflict-suppressed distillation**14:Estimate pixel-wise expert reliability according to expert consistency and confidence.15:Predict routing weights for the Transformer and Mamba experts.16:Fuse reliable expert features and logits according to the routing weights.17:Optimize the student with segmentation loss, multi-scale feature distillation, logit distillation, boundary-aware distillation, and routing supervision.18:**Deployment**19:Remove the Transformer expert, Mamba expert, teacher-side encoder, density prediction branch, expert router, auxiliary heads, and all distillation-related modules.20:Retain only the lightweight CNN student for inference.

## 4. Experiments

### 4.1. Experimental Setup

#### 4.1.1. Datasets

We evaluate the proposed MEKD-UAVSeg on two representative UAV semantic segmentation datasets, namely UAVid and UDD6. UAVid is a high-resolution UAV semantic segmentation benchmark collected from urban street scenes, where UAV images are captured from oblique viewpoints and contain both top-view and side-view visual information. Compared with ground-view urban scene datasets, UAVid presents more challenging characteristics for semantic segmentation, including large-scale variations, dense small objects, complex building structures, fragmented road boundaries, and strong appearance changes caused by different flight altitudes and viewpoints. Following the official protocol, we use the predefined training, validation, and testing splits and evaluate semantic segmentation performance over eight categories, including building, road, tree, low vegetation, moving car, static car, human, and background clutter.

UDD6 is adopted as a complementary UAV semantic segmentation benchmark to further evaluate the robustness and generalization ability of the proposed framework under diverse drone-view scenarios. Compared with UAVid, UDD6 contains more complex aerial scenes with noticeable variations in viewpoints, illumination conditions, object scales, and background layouts. It provides pixel-level annotations for six semantic categories, including Facade, road, vegetation, vehicle, roof, and other. The combination of UAVid and UDD6 enables a more comprehensive evaluation of MEKD-UAVSeg, where UAVid mainly reflects dense urban UAV scene parsing and UDD6 further tests cross-scene adaptability under diverse aerial imaging conditions.

For both datasets, the original high-resolution images are cropped into fixed-size patches during training to fit GPU memory and maintain a consistent input resolution. During testing, all methods are evaluated under the same preprocessing and inference protocol, such as sliding-window or resizing-based evaluation according to the image resolution, to ensure a fair comparison.

#### 4.1.2. Metrics

Following common semantic segmentation protocols, we adopt Intersection over Union (IoU), mean Intersection over Union (mIoU), mean F1-score (mF1), overall accuracy (OA), and the number of inference-stage parameters (Param) as evaluation metrics. For class *k*, IoU and F1-score are defined as

(45) $${\mathrm{IoU}}_{k}=\frac{TP_{k}}{TP_{k}+FP_{k}+FN_{k}},$$

(46) $${F1}_{k}=\frac{2TP_{k}}{2TP_{k}+FP_{k}+FN_{k}},$$

where $TP_{k}$, $FP_{k}$, and $FN_{k}$ denote the numbers of true-positive, false-positive, and false-negative pixels for class *k*, respectively. The mean IoU and mean F1-score are computed by averaging the class-wise scores:

(47) $$\mathrm{mIoU}=\frac{1}{K}\sum_{k=1}^{K}{\mathrm{IoU}}_{k},$$

(48) $$mF1=\frac{1}{K}\sum_{k=1}^{K}{F1}_{k},$$

where *K* denotes the number of evaluated semantic classes. Overall accuracy is calculated as

(49) $$\mathrm{OA}=\frac{\sum_{k=1}^{K}TP_{k}}{N},$$

where *N* is the total number of evaluated pixels.

For UAVid, mIoU and mF1 are computed over all eight semantic categories following the official evaluation protocol. For UDD6, following the commonly used protocol, mIoU is computed over the five foreground categories, including Facade, Road, Vegetation, Vehicle, and Roof, while the Other class is reported in the class-wise results but not included in the foreground mIoU calculation. Param is used to measure the model size at the inference stage. For MEKD-UAVSeg, Param is calculated only for the deployed lightweight CNN student, since the Transformer expert, Mamba expert, teacher-side encoder, density prediction branch, expert router, auxiliary heads, and all distillation-related modules are removed after training.

#### 4.1.3. Optimization Details

**Model and training setup.** All experiments are implemented with PyTorch on NVIDIA RTX 3090 GPUs. Unless otherwise specified, STDC2-FPN is adopted as the default lightweight student. The student decoder follows a lightweight FPN design, where multi-scale encoder features are first projected to a unified channel dimension by 1×1 convolutions and then fused through bilinear upsampling, skip connections, and depthwise separable 3×3 convolutions. The Transformer semantic expert is implemented as a lightweight SegFormer-B1-style branch for high-level semantic aggregation. The Mamba spatial expert contains two directional Mamba blocks, a local depthwise separable convolution path, and four-directional selective scanning. The expert router takes the concatenated Transformer feature, Mamba feature, structure-density map, and UAV hard-region mask as input and predicts spatially adaptive expert weights using lightweight convolutional layers followed by a softmax operation.

Following common UAV semantic segmentation protocols, the original high-resolution images are cropped into 512×512 patches during training. Random horizontal flipping, random scaling in the range of [0.5,2.0], random cropping, and color jittering are adopted for data augmentation. The batch size is set to 16 for both UAVid and UDD6. Unless otherwise specified, the model is trained for 300 epochs, including an 80-epoch warm-up stage and a 220-epoch distillation stage. The uncertainty mask is activated after the first 10 epochs of the distillation stage. We use AdamW as the optimizer with an initial learning rate of $4\times 10^{-4}$ and a weight decay of $1\times 10^{-2}$. A cosine annealing learning rate schedule with 500 warm-up iterations is adopted. The training process follows the two-stage protocol described in Section 3.6. In the warm-up stage, the student encoder, Transformer expert, and Mamba expert are jointly optimized using features from the current student encoder. After warm-up, the student encoder is copied to initialize the teacher-side encoder and is then frozen. In the distillation stage, the teacher-side encoder and the two experts are frozen, while the student network, projection adapters, expert router, and distillation-related modules are optimized.

**Hyperparameter settings.** The main hyperparameters are kept fixed for both UAVid and UDD6. For the segmentation objective, the smoothing constant in the class-balanced weight is set to μ=1.02 to avoid unstable weights for extremely rare classes. The Dice loss weight is set to $\lambda_{\mathrm{dice}}=0.5$, which provides a moderate balance between pixel-wise class-balanced cross-entropy and region-level overlap supervision. We also test $\lambda_{\mathrm{dice}}\in \left\{0.25,0.5,1.0\right\}$ on the validation set, and $\lambda_{\mathrm{dice}}=0.5$ gives the best overall validation mIoU. For expert learning, the two expert losses are assigned equal weights, i.e., $\lambda_{t}=\lambda_{m}=1.0$, while the auxiliary prototype and density losses are given smaller weights, with $\lambda_{\mathrm{proto}}=0.05$ and $\lambda_{\mathrm{den}}=0.1$, respectively. The prototype memory is updated by exponential moving average with momentum η=0.99, and a class prototype is updated only when the number of valid pixels is larger than $n_{\min}=100$. The temperature in the prototype contrastive objective is set to τ=0.2.

For conflict-suppressed distillation, we set the multi-scale feature distillation weight to $\lambda_{\mathrm{ms}}=0.5$, the logit distillation weight to $\lambda_{\mathrm{logit}}=1.0$, the boundary distillation weight to $\lambda_{\mathrm{bd}}=0.2$, and the routing supervision weight to $\lambda_{r}=0.05$. The logit distillation temperature is set to T=4. The hard-region coefficient is set to β=0.5 to avoid over-emphasizing difficult pixels. The consistency and confidence thresholds are updated by an exponential moving average with momentum 0.95. The gate temperatures are set to $\tau_{c}=\tau_{r}=0.2$, and the reliability-target temperature is set to $\tau_{\alpha }=1.0$. The uncertainty mask is disabled during the first 10 epochs of the distillation stage and is then activated to avoid using unstable early predictions.

For the reported mean and standard deviation, each experiment of the proposed method is repeated three times with random seeds 0, 1, and 2. The same seeds are used for the main comparison and ablation variants unless otherwise specified. Competing methods are reproduced under the same dataset split and evaluation protocol when source code is available; otherwise, officially reported results are used.

**Evaluation protocol and training-stage overhead.** During testing, all methods are evaluated under the same preprocessing and inference protocol, without multi-scale testing or test-time augmentation. For efficiency evaluation, all methods are measured under the same hardware environment, input resolution, batch size, and precision setting. Specifically, we use an NVIDIA RTX 3090 GPU and a batch size of 1 for inference timing. Before timing, warm-up iterations are performed to stabilize GPU execution, and FPS and latency are then computed over repeated forward passes with CUDA synchronization. GFLOPs are calculated for a single forward pass under the same input resolution. For the proposed framework, only the deployed STDC2-FPN student is used for efficiency measurement, because the Transformer expert, Mamba expert, teacher-side encoder, density prediction branch, expert router, auxiliary heads, projection adapters, and all distillation-related modules are removed after training.

The additional computational cost of the proposed framework mainly occurs in the offline training stage. Compared with the student-only baseline, the proposed framework introduces the Transformer semantic expert, the Mamba spatial expert, the teacher-side encoder, the density prediction branch, the expert router, and several distillation losses during training. These modules increase training memory consumption and optimization cost, but they are not involved in inference. Therefore, the training-stage overhead is exchanged for a stronger deployed student, while the final network keeps exactly the same inference architecture as the STDC2-FPN baseline. Accordingly, the reported inference parameters, GFLOPs, FPS, and latency exclude all training-only expert and distillation modules.

### 4.2. Comparison Against State-of-the-Art

#### 4.2.1. Results on the UAVid Dataset

Table 1 presents the quantitative comparison on the UAVid dataset. The compared methods cover CNN-based, Transformer-based, Mamba-based, and hybrid architectures. MEKD-UAVSeg achieves the best overall performance, with 69.17±0.11% mIoU, 80.35±0.12% mF1, and 86.62±0.09% OA over multiple runs. Compared with UAV-FAENet, the strongest competing method in terms of mIoU, MEKD-UAVSeg improves mIoU from 68.84% to 69.17%, mF1 from 79.97% to 80.35%, and OA from 86.48% to 86.62%. Although the gains over the strongest baseline are moderate, the small standard deviations indicate that the proposed method performs stably across different runs.

From the class-wise results, MEKD-UAVSeg obtains the best IoU on Building and LowVeg, reaching 87.92% and 65.10%, respectively. These categories are usually affected by complex spatial layouts, scale variations, and ambiguous boundaries in UAV imagery. The proposed method also achieves the second-best performance on Road, Tree, MovingCar, StaticCar, and Human, with IoU scores of 83.05%, 79.41%, 77.85%, 61.28%, and 30.65%, respectively. Although DC-Swin performs best on Tree and Clutter, and MANet obtains the highest IoU on StaticCar, MEKD-UAVSeg ranks first in all overall metrics. This indicates that the proposed framework provides balanced improvements across large-area regions, small objects, and boundary-sensitive categories.

In terms of model complexity, MEKD-UAVSeg uses 16.45 M inference parameters, which are lower than UAV-FAENet with 19.17 M parameters and much smaller than RS3Mamba and DC-Swin with 43.32 M and 45.63 M parameters, respectively. Compared with DC-Swin and RS3Mamba, MEKD-UAVSeg improves mIoU by 1.47 and 1.02 percentage points while using substantially fewer inference parameters. Since the Transformer and Mamba experts are used only during training, the deployed model does not introduce additional expert branches at inference time. Therefore, the performance gain mainly comes from more effective knowledge transfer rather than increased inference-stage model capacity.

**Figure 3:**
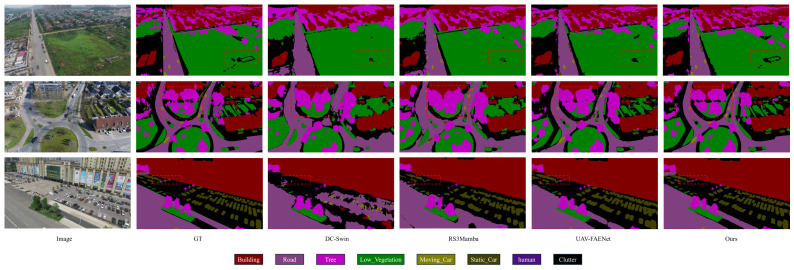
Qualitative comparison on the UAVid dataset. From left to right, each row shows the input image, ground truth, DC-Swin, RS3Mamba, UAV-FAENet, and the proposed MEKD-UAVSeg. The dashed boxes indicate challenging regions where MEKD-UAVSeg better preserves road boundaries, vegetation structures, small vehicles, and cluttered urban details.

Figure 3 presents qualitative comparisons on the UAVid dataset. Compared with DC-Swin, RS3Mamba, and UAV-FAENet, MEKD-UAVSeg produces more spatially consistent predictions around road regions, vegetation areas, building boundaries, and vehicle-related regions. The red dashed boxes highlight challenging areas where the proposed method better preserves local structures and suppresses isolated misclassified regions. These visual results are consistent with the quantitative improvements in Table 1, showing that heterogeneous expert distillation helps the lightweight CNN student capture both semantic consistency and spatial structural details.

#### 4.2.2. Results on the UDD6 Dataset

Table 2 reports the quantitative comparison on the UDD6 dataset. Following the common evaluation protocol of UDD6, mIoU is computed over the five foreground categories, including Facade, Road, Vegetation, Vehicle, and Roof, while the Other class is reported separately. MEKD-UAVSeg achieves the best overall performance, with 80.68±0.10% mIoU, 88.95±0.08% mF1, and 89.12±0.07% OA over multiple runs. Compared with UAV-FAENet, the strongest competing method in terms of mIoU, MEKD-UAVSeg improves mIoU from 80.11% to 80.68%, mF1 from 88.74% to 88.95%, and OA from 88.80% to 89.12%. Although the gain over the strongest baseline is moderate, the small standard deviations indicate stable performance on UDD6, which contains different scene layouts and object distributions from UAVid.

From the class-wise results, MEKD-UAVSeg obtains the best IoU on Facade, Road, and Vehicle, reaching 76.52%, 73.85%, and 73.60%, respectively. These categories usually involve complex structures, large appearance variations, or small object regions under UAV viewpoints. The proposed method also achieves the second-best performance on Roof and Other, with IoU scores of 89.85% and 64.90%, which are close to the best results. Although ABCNet obtains the highest IoU on Vegetation, MEKD-UAVSeg remains competitive in this category. This distribution suggests that the proposed method does not improve the overall score by favoring a single class but provides balanced performance across dominant foreground regions, small objects, and boundary-sensitive categories.

In terms of model complexity, MEKD-UAVSeg uses only 16.45M inference parameters, which is lower than UAV-FAENet with 19.17M parameters and much smaller than DC-Swin and RS3Mamba with 45.63M and 43.32M parameters, respectively. Compared with DC-Swin and RS3Mamba, MEKD-UAVSeg achieves higher mIoU with a substantially smaller deployed model. Since the Transformer and Mamba experts are used only during training and removed after distillation, the performance gain is obtained without increasing inference-stage model complexity. This property is important for practical UAV semantic segmentation under limited computational resources.

**Figure 4:**
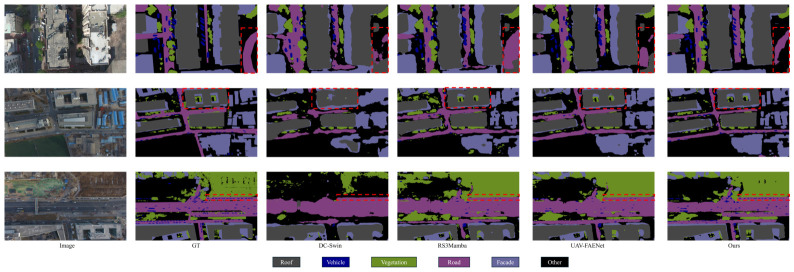
Qualitative comparison on the UDD6 dataset. From left to right, each row shows the input image, ground truth, DC-Swin, RS3Mamba, UAV-FAENet, and the proposed MEKD-UAVSeg. Compared with representative Transformer-, Mamba-, and hybrid-based methods, MEKD-UAVSeg better preserves structural boundaries and local object regions, such as roads, roofs, vehicles, vegetation, and facades. Red dashed boxes indicate challenging regions with small objects, ambiguous appearances, or complex spatial layouts.

Figure 4 provides qualitative comparisons on the UDD6 dataset. Compared with DC-Swin, RS3Mamba, and UAV-FAENet, MEKD-UAVSeg produces more complete and coherent predictions for roads, roofs, facades, vegetation, and vehicle regions. It better preserves structural boundaries in roof–road and vegetation–road transition areas, while reducing local misclassification in visually ambiguous regions. These observations are consistent with the quantitative results in Table 2, further showing that the training-only Transformer and Mamba experts provide complementary guidance for the deployed CNN student.

It should also be noted that OA is dominated by large-area categories, such as road, building, roof, vegetation, and facade. Therefore, a method may obtain competitive OA even when its performance on small objects, rare categories, or boundary-sensitive regions is limited. In contrast, mIoU and mF1 provide more balanced class-wise evaluation. The proposed method improves mIoU and hard-region metrics while maintaining competitive OA, indicating that the gain does not simply come from dominant classes but also from improved recognition of small-object and boundary-sensitive regions.

#### 4.2.3. Comparison with Existing Distillation Strategies

To further examine whether the performance gain comes from the proposed distillation strategy rather than only from the student architecture, we compare MEKD-UAVSeg with representative knowledge distillation methods. All methods use the same STDC2-FPN student, dataset split, training resolution, and inference protocol. During inference, only the student network is retained for all methods, and thus the inference-stage model size is identical across different distillation strategies and omitted from the table.

Table 3 shows that existing distillation strategies improve the lightweight student to different extents. Vanilla KD provides consistent gains over the student-only baseline, while CWD and CIRKD further improve the results by transferring channel-wise or relational knowledge. Boundary-oriented methods, such as BPKD and BRD, achieve stronger performance because they emphasize spatially sensitive regions that frequently appear in UAV imagery.

MEKD-UAVSeg achieves the best results on both datasets. On UAVid, it improves the student-only baseline from 66.45%, 77.82%, and 84.90% to 69.17%, 80.35%, and 86.62% in mIoU, mF1, and OA, respectively. Compared with the strongest distillation baseline, BPKD, MEKD-UAVSeg further improves mIoU by 0.82 percentage points, mF1 by 0.85 percentage points, and OA by 0.57 percentage points. On UDD6, it improves mIoU from 77.50% to 80.68% over the student-only baseline and outperforms BPKD by 0.83 percentage points in mIoU.

These results indicate that generic distillation strategies are useful but still limited for UAV semantic segmentation. Existing methods do not explicitly distinguish region-level semantics, direction-sensitive spatial structures, and spatially varying expert reliability. In contrast, MEKD-UAVSeg combines heterogeneous training-only experts, UAV-aware priors, and conflict-suppressed routing, allowing the student to receive more reliable supervision in small-object, boundary, and structurally complex regions. Since all methods share the same deployed student, the improvement mainly comes from the proposed distillation strategy rather than increased inference-stage capacity.

#### 4.2.4. Accuracy–Efficiency Trade-Off for UAV-Borne Visual Sensing

In addition to segmentation accuracy, deployment efficiency is an important factor for UAV semantic segmentation. Table 4 reports the efficiency comparison on the UAVid dataset in terms of FPS, GFLOPs, latency, parameters, mIoU, and mF1. Overall, MEKD-UAVSeg achieves a favorable balance between segmentation accuracy and inference efficiency. Compared with UAV-FAENet, which is the strongest competing method in Table 4, MEKD-UAVSeg improves mIoU from 68.84% to 69.17% and mF1 from 79.97% to 80.35%. Meanwhile, it reduces GFLOPs from 76.9 to 58.4, decreases latency from 19.2 ms to 14.6 ms, reduces parameters from 19.17M to 16.45M, and improves FPS from 52.0 to 68.5. These results indicate that the proposed method does not improve accuracy by increasing inference-stage complexity.

Compared with heavier Transformer- and Mamba-based methods, MEKD-UAVSeg also shows clear deployment advantages. For example, DC-Swin requires 170.3 GFLOPs and reaches 23.5 FPS, while RS3Mamba requires 226.0 GFLOPs and reaches 17.7 FPS. In contrast, MEKD-UAVSeg only requires 58.4 GFLOPs and achieves 68.5 FPS, while obtaining higher mIoU than both methods. Although several faster methods, such as ABCNet, UNetFormer, and MANet, achieve higher FPS, their segmentation accuracy is lower than that of MEKD-UAVSeg. This suggests that MEKD-UAVSeg provides a better accuracy–efficiency trade-off among high-performing UAV segmentation models.

**Figure 5:**
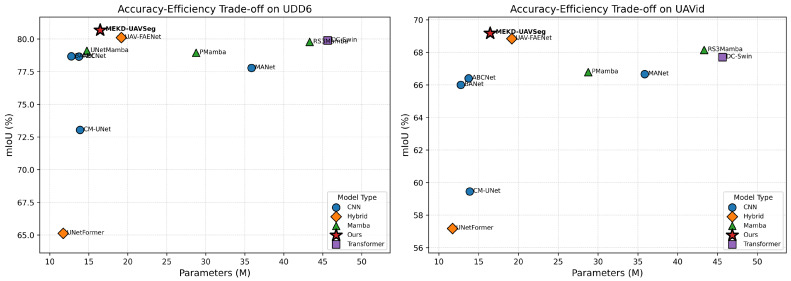
Accuracy–efficiency trade-off on UDD6 and UAVid. The horizontal axis denotes the number of inference-stage parameters, and the vertical axis denotes mean Intersection over Union (mIoU). Each point represents a segmentation method. MEKD-UAVSeg achieves higher segmentation accuracy with a compact inference model because the Transformer and Mamba experts are used only during training and removed after distillation.

Figure 5 further illustrates the accuracy–efficiency trade-off by comparing mIoU with the number of inference-stage parameters on UAVid and UDD6. The proposed MEKD-UAVSeg achieves the best mIoU on both datasets while maintaining a compact deployed model with 16.45M parameters. This favorable trade-off is mainly attributed to the training-only expert distillation design. During training, the Transformer semantic expert and Mamba spatial expert provide complementary semantic and spatial guidance for the CNN student. During inference, all expert branches, routing modules, density prediction branches, and distillation-related components are removed. Therefore, the deployed model keeps exactly the same inference structure as the lightweight CNN student, and the performance gain comes from more effective knowledge transfer rather than increased inference-stage model capacity.

The efficiency results further indicate the suitability of MEKD-UAVSeg for UAV-borne visual sensing. In practical aerial sensing applications, semantic perception modules are often required to process high-resolution visual observations under limited onboard computation, memory, and latency budgets. The proposed framework improves the deployed CNN student through offline expert distillation rather than adding heavy Transformer or Mamba modules to the inference branch. As shown in Table 4, MEKD-UAVSeg achieves 68.5 FPS, 58.4 GFLOPs, and 14.6 ms latency under the adopted evaluation setting, while maintaining the best mIoU among the compared methods. More importantly, the reported inference parameters, GFLOPs, FPS, and latency are measured only on the final CNN student, because the Transformer semantic expert, Mamba spatial expert, teacher-side encoder, density prediction branch, expert router, auxiliary heads, projection adapters, and distillation losses are all removed after training. Therefore, the proposed method provides a favorable accuracy–efficiency trade-off for deployment-constrained UAV visual sensor data processing scenarios where high-capacity training guidance is desired but inference-stage complexity must remain low. Since the current efficiency evaluation is conducted on an RTX 3090 for fair comparison with existing methods, the reported FPS should be interpreted as a relative efficiency indicator rather than a complete onboard deployment validation. Real-time performance and power consumption on embedded UAV processors, such as Jetson Xavier NX or Jetson Orin, will be further evaluated in future work.

#### 4.2.5. Hard-Region Analysis

Since the proposed framework is designed to improve difficult UAV regions, we further analyze its behavior on small objects, rare categories, and boundary-sensitive regions. Compared with general scene segmentation, UAV imagery contains many objects with limited pixel areas, such as vehicles and pedestrians, as well as fragmented boundaries around roads, roofs, vegetation, and building contours. These regions are particularly prone to inconsistent expert responses and negative knowledge transfer.

To evaluate this aspect, we report hard-region metrics in Table 5. For UAVid, the small-object subset includes MovingCar, StaticCar, and Human, while the rare-class subset includes StaticCar, Human, and Clutter. For UDD6, Vehicle IoU is used as the representative small-object indicator, and foreground mIoU is computed over the five foreground categories, including Facade, Road, Vegetation, Vehicle, and Roof. Boundary F-score is computed by matching the predicted and ground-truth semantic boundaries within a fixed tolerance region. This evaluation complements the overall mIoU by focusing on the regions where UAV segmentation errors are most likely to occur.

The results show that MEKD-UAVSeg provides consistent improvements in hard UAV regions. On UAVid, MEKD-UAVSeg achieves the best small-object mIoU and rare-class mIoU, indicating that UAV-aware hard-region priors help the student focus on visually small and under-represented categories that are easily overwhelmed by background pixels. Compared with UAV-FAENet and RS3Mamba, the proposed method also improves Boundary F-score, suggesting that the Mamba spatial expert and boundary-aware distillation provide useful structural guidance for fragmented contours and elongated regions.

On UDD6, MEKD-UAVSeg obtains the highest Vehicle IoU, Boundary F-score, and foreground mIoU. The improvement in Vehicle IoU indicates that the proposed framework benefits object-level recognition under UAV viewpoints, while the gain in Boundary F-score demonstrates its advantage in preserving roof–road, facade–road, and vegetation–road transition regions. These results are consistent with the motivation of conflict-suppressed heterogeneous expert distillation: reliable expert knowledge should be selectively transferred according to spatially varying difficulty and expert reliability. In ambiguous regions where semantic and spatial experts may produce inconsistent responses, the proposed reliability routing and consistency–confidence gating reduce negative transfer and help the deployed CNN student learn more robust representations.

### 4.3. Ablation Study

To verify the effectiveness of the proposed MEKD-UAVSeg, we conduct ablation experiments on UAVid and UDD6. All ablation variants adopt the same default configuration unless otherwise specified, where STDC2-FPN is used as the deployed CNN student, a SegFormer-B1-style branch is used as the Transformer semantic expert, and a two-block directional Mamba branch is used as the spatial expert. The Transformer and Mamba experts are only used during training and are removed during inference. Therefore, all MEKD variants keep the same deployed inference structure as the CNN student.

#### 4.3.1. Main Component Analysis

Table 6 presents the progressive component ablation results on UAVid and UDD6. All variants share the same STDC2-FPN student during inference, and the additional expert branches and distillation modules are only used in the training stage. Therefore, the performance differences mainly reflect the contribution of each training component rather than changes in the deployed inference architecture.

The baseline STDC2-FPN student obtains 66.45% mIoU, 77.82% mF1, and 84.90% OA on UAVid, and 77.50% mIoU, 87.10% mF1, and 87.20% OA on UDD6. After introducing the heterogeneous Transformer–Mamba experts, the performance increases to 67.12% and 78.35% mIoU on UAVid and UDD6, respectively. This improvement shows that the semantic modeling ability of the Transformer expert and the spatial dependency modeling ability of the Mamba expert provide useful complementary supervision for the lightweight student. When multi-scale cross-branch distillation is further introduced, the mIoU improves from 67.12% to 67.68% on UAVid and from 78.35% to 79.05% on UDD6. This indicates that aligning expert knowledge at multiple feature levels is more effective than relying only on single-level supervision, especially for UAV scenes with large-scale variations. The UAV-aware density and hard-region priors further improve the mIoU to 68.15% on UAVid and 79.52% on UDD6. The consistent gains suggest that explicitly emphasizing dense small-object regions and difficult pixels helps the student focus on challenging UAV-specific patterns. The conflict-suppressed reliability routing module also brings clear improvements, increasing the mIoU from 68.15% to 68.54% on UAVid and from 79.52% to 80.05% on UDD6. This demonstrates the necessity of selectively transferring expert knowledge rather than treating all expert responses as equally reliable. After adding the consistency and confidence gates, the model reaches 68.92% mIoU on UAVid and 80.40% mIoU on UDD6, showing that suppressing uncertain or inconsistent supervision can further stabilize the distillation process.

With all components integrated, MEKD-UAVSeg achieves 69.17% mIoU, 80.35% mF1, and 86.62% OA on UAVid, and 80.68% mIoU, 88.95% mF1, and 89.12% OA on UDD6. Compared with the baseline student, the full model improves mIoU by 2.72 percentage points on UAVid and 3.18 percentage points on UDD6, while keeping the same inference-time student structure. These results verify that the proposed framework improves the representation ability of the lightweight student through training-stage heterogeneous expert guidance, multi-scale distillation, UAV-aware priors, and reliability-aware knowledge transfer, without increasing the deployment complexity.

The component ablation results also indicate which visual failure modes are affected by different modules. The heterogeneous Transformer–Mamba experts mainly improve the complementarity between semantic consistency and spatial continuity. The Transformer expert benefits large-area semantic discrimination, such as roads, buildings, roofs, and vegetation regions, while the Mamba expert helps preserve elongated and direction-sensitive structures. Multi-scale cross-branch distillation further improves scale-aware transfer, which is important for UAV images containing both large background regions and small objects. UAV-aware density and hard-region priors provide additional gains by emphasizing small-object, boundary-rich, rare-class, and uncertain regions. Conflict-suppressed reliability routing and consistency–confidence gates further reduce negative transfer by weakening unreliable supervision when the experts disagree or when their confidence is low. Therefore, the cumulative improvement is not only reflected in overall mIoU but also corresponds to practical visual improvements in roads, roofs, vehicles, boundaries, cluttered regions, and scale-varying objects.

#### 4.3.2. Analysis of Heterogeneous Experts

To further verify whether the performance gain comes from the heterogeneous nature of the expert design, we conduct an expert-configuration ablation study in Table 7. In this experiment, all variants use the same CNN student and training setting. For dual-expert variants, the fusion and distillation design is kept identical, and only the expert architecture is changed. Therefore, this ablation isolates the influence of different expert configurations.

Compared with the student-only baseline, both single-expert variants bring consistent improvements on the two datasets. On UAVid, the single Transformer expert improves the mIoU from 66.45% to 66.98%, while the single Mamba expert improves it to 66.85%. On UDD6, the single Transformer and single Mamba experts improve the mIoU from 77.50% to 78.10% and 78.25%, respectively. These results indicate that both semantic-oriented Transformer modeling and spatial-dependency-oriented Mamba modeling can provide useful supervision for the lightweight CNN student. Using two experts of the same architecture further improves the performance. Specifically, dual Transformer experts achieve 67.45% mIoU on UAVid and 78.60% mIoU on UDD6, while dual Mamba experts achieve 67.30% and 78.75% mIoU, respectively. This shows that increasing the amount of expert supervision is beneficial. However, the improvement obtained by homogeneous experts is still limited, suggesting that using multiple experts with similar modeling biases may introduce redundant supervision. In contrast, the Transformer + Mamba configuration achieves the best performance on both datasets, reaching 67.82% mIoU, 79.05% mF1, and 85.80% OA on UAVid, and 79.15% mIoU, 88.35% mF1, and 88.40% OA on UDD6. Compared with the stronger homogeneous dual-expert setting, the heterogeneous configuration improves mIoU by 0.37 percentage points on UAVid and 0.40 percentage points on UDD6. These consistent gains demonstrate that the Transformer and Mamba experts provide complementary knowledge rather than merely increasing the number of teacher branches.

The results also reveal different tendencies of the two expert types. On UAVid, the Transformer expert performs slightly better than the Mamba expert, which may be related to its stronger semantic representation ability for complex urban categories. On UDD6, the Mamba expert performs slightly better, suggesting that spatial dependency modeling is beneficial for scenes with structured regions such as roads, roofs, and facades. By combining the two types of experts, the proposed framework can exploit both high-level semantic discrimination and spatial structural modeling, leading to more effective knowledge transfer to the CNN student.

To further respond to the concern about whether Mamba is necessary as the spatial expert, we conduct an additional comparison under the full MEKD-UAVSeg framework (Table 8). Different from Table 7, which isolates expert configurations before all components are integrated, this experiment keeps the full distillation pipeline unchanged and replaces only the spatial expert branch.

The Mamba spatial expert obtains the best results among the compared spatial expert designs under our setting. Compared with the dilated CNN expert and the lightweight attention expert, it improves mIoU by 0.31 and 0.20 percentage points on UAVid, and by 0.32 and 0.23 percentage points on UDD6, respectively. These results suggest that the selective-scan spatial bias is useful for structured UAV regions. Nevertheless, the improvement remains moderate, indicating that the main contribution of MEKD-UAVSeg lies in conflict-suppressed heterogeneous expert distillation rather than in claiming Mamba as the only effective spatial expert.

#### 4.3.3. Ablation on Training Protocol

Table 9 analyzes the influence of different training protocols. In this study, all variants use the same STDC2-FPN student for inference, and the differences only lie in whether student warm-up, expert knowledge distillation, and the frozen teacher-side source are adopted. The warm-up-only variant is trained with the same overall training budget but removes expert distillation, so that the effect of warm-up can be separated from the contribution of knowledge transfer. The frozen source denotes the teacher-side feature source obtained after the warm-up stage and kept fixed during the subsequent distillation stage.

The student-only baseline achieves 66.45% mIoU on UAVid and 77.50% mIoU on UDD6. When direct KD is applied without warm-up, the performance improves to 67.52% and 79.20% mIoU on the two datasets, respectively. This confirms that the expert branches can provide useful supervision to the lightweight student. However, direct distillation from the beginning of training may introduce unstable guidance because the student representation is still immature at early iterations. The warm-up-only setting brings limited but consistent improvements over the student-only baseline, increasing the mIoU from 66.45% to 66.85% on UAVid and from 77.50% to 78.10% on UDD6. This indicates that the warm-up stage can improve the initialization of the student model, but it is insufficient to fully exploit the knowledge from heterogeneous experts. When expert KD is performed after warm-up, but the teacher-side source is still updated online, the mIoU further increases to 68.45% on UAVid and 80.05% on UDD6. These results show that warm-up and distillation are complementary, and a more stable student representation can benefit the subsequent knowledge transfer process.

As shown in Table 10, the proposed two-stage KD achieves the best performance on both datasets, with 69.17% mIoU, 80.35% mF1, and 86.62% OA on UAVid, and 80.68% mIoU, 88.95% mF1, and 89.12% OA on UDD6. Compared with warm-up + online KD, the proposed strategy improves mIoU by 0.72 percentage points on UAVid and 0.63 percentage points on UDD6. This demonstrates that freezing the teacher-side feature source after warm-up can reduce representation drift during distillation and provide more stable expert guidance. Overall, the results verify that the proposed training protocol is necessary for effectively transferring heterogeneous expert knowledge to the deployed CNN student.

The feature-stability analysis supports the motivation of freezing the teacher-side encoder after warm-up. In the online KD setting, the teacher-side features and student features change simultaneously, leading to lower feature similarity and larger fluctuation. In contrast, the proposed two-stage KD provides a stable teacher-side source for the Transformer and Mamba experts, which improves teacher–student feature alignment and leads to better distillation performance.

### 4.4. Sensitivity Analysis

As shown in Table 11, we further analyze the sensitivity of several important hyperparameters in the proposed framework, including the uncertainty-mask activation epoch, the consistency–confidence gate temperatures, and the Dice loss weight. These analyses examine whether the performance gain is caused by fragile hyperparameter choices or by the overall design of reliability-aware heterogeneous expert distillation.

Activating the uncertainty mask from the beginning introduces noisy student predictions into the hard-region prior, which may reduce the reliability of early distillation. Activating it too late weakens its contribution to reliability-aware knowledge transfer. The default setting $e_{\mathrm{act}}=10$ provides a stable balance between avoiding early noise and exploiting uncertainty-aware hard-region cues during the main distillation stage.

The performance remains stable around the default setting. A very small temperature makes the gate too sharp and may suppress useful expert knowledge, while a large temperature makes the gate too smooth and weakens conflict suppression. As shown in Table 12, the setting $\tau_{c}=\tau_{r}=0.2$ gives a good trade-off between selective knowledge transfer and training stability. In addition, the asymmetric settings (0.2,0.1) and (0.1,0.2) produce slightly lower results than the shared-temperature setting, indicating that the threshold-normalized consistency and confidence gates can be controlled by the same transition temperature without obvious performance loss.

The model is not highly sensitive to the Dice loss weight within the tested range. As shown in Table 13, a small value weakens region-level overlap supervision, while a large value may over-emphasize region consistency and slightly reduce class-balanced pixel discrimination. The setting $\lambda_{\mathrm{dice}}=0.5$ provides the best validation performance on both datasets and is therefore adopted as the default value.

## 5. Discussion

The experimental results indicate that the main value of MEKD-UAVSeg lies in improving a compact UAV visual sensing model through training-only expert guidance rather than through inference-time architectural expansion. This distinguishes our method from many Transformer- and Mamba-based segmentation networks that directly insert heavy global-context modules into the deployed model. In contrast, MEKD-UAVSeg uses Transformer and Mamba experts only during offline optimization and removes all expert branches after distillation. Therefore, the deployed model retains the efficiency of the CNN student while benefiting from complementary semantic and spatial supervision during training.

Compared with existing UAV and remote sensing segmentation methods, the proposed framework addresses a different aspect of the accuracy–efficiency trade-off. Methods such as UNetFormer, DC-Swin, RS3Mamba, PMamba, and UAV-FAENet improve representation ability by modifying the inference network. These designs are effective, but their computational cost or parameter size may increase when stronger context modeling is introduced. Knowledge distillation methods such as CWD, CIRKD, BPKD, and BRD improve compact students, but they usually rely on a single teacher or use boundary cues mainly as additional losses. MEKD-UAVSeg combines heterogeneous training-only experts, UAV-aware priors, and reliability-aware routing, allowing the student to receive spatially selective knowledge in small-object, boundary-rich, and ambiguous UAV regions.

The results also show that the improvement is moderate but consistent. This is expected because the deployed student is intentionally kept compact and no additional inference-time expert module is retained. Therefore, the contribution of MEKD-UAVSeg should be interpreted as a deployment-oriented training strategy rather than a heavy, high-capacity segmentation architecture. The Mamba expert provides complementary spatial supervision, but we do not claim that it is the only possible or universally optimal spatial expert. The additional comparison with dilated CNN and lightweight attention experts further shows that alternative spatial expert designs can also provide useful guidance, although the Mamba expert gives the best results under the current setting.

Several limitations remain. First, the UAV-aware priors rely on pixel-level annotations to construct boundary, small-object, rare-class, and uncertainty cues during training. Their effectiveness may therefore be affected by annotation noise or inconsistent labeling of small objects and boundaries. Second, the current evaluation is conducted on UAVid and UDD6. Although these datasets contain diverse UAV scenes, further validation is needed under night scenes, adverse weather, larger altitude variations, and cross-sensor domain shifts. Third, the current efficiency evaluation is measured on an RTX 3090 for fair comparison with prior methods. Embedded deployment on UAV processors such as Jetson Xavier NX or Jetson Orin, including latency and power consumption, remains to be further evaluated. Finally, the training stage is more complex than student-only optimization because the Transformer expert, Mamba expert, teacher-side encoder, density branch, router, and distillation losses are used offline. Future work will investigate hardware-aware deployment, cheaper spatial expert designs, cross-domain robustness, and extension to infrared, multispectral, event-camera, and video-based UAV perception.

## 6. Conclusions

This paper presented MEKD-UAVSeg, a lightweight semantic perception framework for UAV-borne visual sensor data based on conflict-suppressed heterogeneous expert distillation. The framework improves a compact CNN student by using Transformer semantic guidance and Mamba spatial guidance only during training. UAV-aware priors and reliability-aware routing are further introduced to reduce negative transfer in small-object, boundary-rich, rare-class, and ambiguous aerial regions. Since all expert branches and distillation modules are discarded after training, the deployed model remains a lightweight CNN without expert-induced inference complexity.

Experiments on UAVid and UDD6 demonstrate that the proposed framework achieves a favorable accuracy–efficiency trade-off compared with representative CNN-, Transformer-, Mamba-, hybrid-, and distillation-based methods. The ablation results verify the contributions of heterogeneous expert guidance, UAV-aware priors, conflict-suppressed routing, the two-stage training protocol, and key hyperparameter settings. Future work will focus on embedded hardware validation, power-aware UAV deployment, more efficient spatial expert alternatives, cross-domain UAV sensing under adverse weather and night conditions, and extension to multimodal UAV sensors such as infrared, multispectral, and event cameras.

## Figures and Tables

**Table 1:** Quantitative comparison of different methods on the UAVid dataset. For the proposed method, we report the mean and standard deviation over multiple runs. For competing methods, we report reproduced or officially reported results under the same evaluation protocol. The best and second-best segmentation results are highlighted in bold and underlined, respectively.

Method	Type	IoU (%)								mIoU (%)	mF1 (%)	OA (%)	Param (M)
		Building	Road	Tree	LowVeg	MovingCar	StaticCar	Human	Clutter				
ABCNet [7]	CNN	85.77	80.92	77.88	62.46	74.12	53.33	29.29	66.14	66.40	78.13	85.48	13.74
BANet [45]	CNN	83.90	81.48	78.51	62.18	77.44	52.01	29.51	64.58	66.00	77.86	84.99	12.76
MANet [46]	CNN	84.42	80.17	78.75	62.39	68.73	**62.73**	29.40	66.17	66.66	78.50	85.19	35.86
DC-Swin [47]	Transformer	87.64	82.49	**79.78**	64.64	76.02	53.62	29.72	**68.43**	67.70	79.01	86.58	45.63
UNetFormer [8]	Hybrid	75.38	75.36	72.11	56.95	64.57	35.89	20.03	55.86	57.18	70.42	79.75	11.72
CM-UNet [48]	CNN	79.05	78.71	73.55	56.85	69.71	33.64	24.64	59.30	59.45	72.24	81.59	13.88
RS3Mamba [39]	Mamba	86.54	81.83	79.03	63.10	75.79	60.42	30.36	67.22	68.15	79.51	86.01	43.32
PMamba [49]	Mamba	85.93	81.20	78.66	62.38	73.95	56.45	28.96	66.48	66.79	78.43	85.59	28.76
UAV-FAENet [31]	Hybrid	87.37	**83.20**	79.26	63.54	**78.02**	59.53	**30.98**	68.25	68.84	79.97	86.48	19.17
MEKD-UAVSeg (Ours)	Hybrid	**87.92**	83.05	79.41	**65.10**	77.85	61.28	30.65	68.10	$69.17_{\pm 0.11}$	$80.35_{\pm 0.12}$	$86.62_{\pm 0.09}$	16.45

**Table 2:** Quantitative comparison results on the UDD6 dataset. For the proposed method, we report the mean and standard deviation over multiple runs. For competing methods, we report reproduced or officially reported results under the same evaluation protocol. The best and second-best segmentation results are highlighted in bold and underlined, respectively.

Methods	Type	IoU (%)						mIoU (%)	mF1 (%)	OA (%)	Param (M)
		Facade	Road	Vegetation	Vehicle	Roof	Other				
ABCNet [7]	CNN	73.10	70.05	**89.79**	71.64	88.68	63.27	78.65	87.76	88.13	13.74
BANet [45]	CNN	73.72	70.59	89.52	71.75	87.77	62.96	78.67	87.83	88.03	12.76
MANet [46]	CNN	72.53	68.84	89.49	70.37	87.64	62.58	77.78	87.22	87.58	35.86
DC-Swin [47]	Transformer	75.50	73.03	89.72	72.08	89.14	**64.95**	79.89	88.61	88.95	45.63
UNetFormer [8]	Hybrid	55.87	58.40	87.16	48.16	76.08	50.20	65.13	78.00	80.39	11.72
CM-UNet [48]	CNN	64.76	66.26	88.88	60.38	84.94	55.71	73.04	83.91	85.06	13.88
RS3Mamba [39]	Mamba	75.46	72.14	89.68	72.01	89.57	64.44	79.77	88.52	88.87	43.32
PMamba [49]	Mamba	74.30	71.04	89.31	70.97	89.06	63.79	78.94	87.98	88.38	28.76
UNetMamba [41]	Mamba	73.52	71.17	89.33	72.71	88.78	63.22	79.10	88.10	88.19	14.75
UAV-FAENet [31]	Hybrid	75.39	72.43	89.58	73.27	**89.89**	63.78	80.11	88.74	88.80	19.17
MEKD-UAVSeg (Ours)	Hybrid	**76.52**	**73.85**	89.60	**73.60**	89.85	64.90	$80.68_{\pm 0.10}$	$88.95_{\pm 0.08}$	$89.12_{\pm 0.07}$	16.45

**Table 3:** Comparison with existing distillation strategies on UAVid and UDD6. All methods use the same STDC2-FPN student for inference. The best and second-best results are highlighted in bold and underlined, respectively.

Method	UAVid			UDD6		
	mIoU	mF1	OA	mIoU	mF1	OA
Student only	66.45	77.82	84.90	77.50	87.10	87.20
Vanilla KD [50]	67.15	78.40	85.35	78.30	87.65	87.75
CWD [15]	67.68	78.95	85.70	78.90	88.05	88.25
CIRKD [16]	67.42	78.70	85.55	78.65	87.85	88.00
BPKD [22]	68.35	79.50	86.05	79.85	88.50	88.65
BRD [23]	68.05	79.25	85.90	79.50	88.30	88.45
MEKD-UAVSeg (Ours)	**69.17**	**80.35**	**86.62**	**80.68**	**88.95**	**89.12**

**Table 4:** Efficiency comparison on the UAVid dataset. FPS, GFLOPs, latency, parameters, mIoU, and mF1 are reported to evaluate the accuracy–efficiency trade-off of different methods. All efficiency results are measured under the same GPU, input resolution, batch size, precision setting, warm-up protocol, and repeated forward-pass protocol. For the proposed framework, all training-only expert branches and distillation modules are removed during measurement, and only the deployed CNN student is retained.

Method	FPS ↑	GFLOPs ↓	Latency (ms) ↓	Param (M) ↓	mIoU ↑	mF1 ↑
ABCNet [7]	102.2	62.9	9.8	13.74	66.40	78.13
BANet [45]	37.3	107.2	26.8	12.76	66.00	77.86
MANet [46]	75.6	51.7	13.2	35.86	66.66	78.50
DC-Swin [47]	23.5	170.3	42.6	45.63	67.70	79.01
UNetFormer [8]	115.6	46.9	8.7	11.72	57.18	70.42
CM-UNet [48]	47.7	83.8	21.0	13.88	59.45	72.24
RS3Mamba [39]	17.7	226.0	56.5	43.32	68.15	79.51
PMamba [49]	49.9	80.2	20.0	28.76	66.79	78.43
UAV-FAENet [31]	52.0	76.9	19.2	19.17	68.84	79.97
MEKD-UAVSeg (Ours)	68.5	58.4	14.6	16.45	69.17	80.35

**Table 5:** Hard-region evaluation on UAVid and UDD6. Small-object mIoU, rare-class mIoU, and boundary F-score are reported to evaluate the effectiveness of the proposed framework in difficult UAV regions. For UAVid, small-object mIoU is computed over MovingCar, StaticCar, and Human, while rare-class mIoU is computed over StaticCar, Human, and Clutter. **Bold** and underlined values indicate the best and second-best results, respectively.

Method	UAVid			UDD6		
	Small-Object mIoU	Rare-Class mIoU	Boundary F-Score	Vehicle IoU	Boundary F-Score	Foreground mIoU
Student only	51.20	48.35	63.40	68.20	70.55	77.50
UAV-FAENet [31]	56.18	52.92	68.85	73.27	74.60	80.11
RS3Mamba [39]	55.52	52.67	68.20	72.01	73.85	79.77
MEKD-UAVSeg	**56.59**	**53.34**	**70.15**	**73.60**	**75.42**	**80.68**

**Table 6:** Component ablation of MEKD-UAVSeg on UAVid and UDD6. All variants use the same STDC2-FPN student during inference. **Bold** values indicate the best results.

Configuration	UAVid			UDD6		
	mIoU	mF1	OA	mIoU	mF1	OA
STDC2-FPN student	66.45	77.82	84.90	77.50	87.10	87.20
+ Heterogeneous Transformer–Mamba experts	67.12	78.45	85.35	78.35	87.65	87.75
+ Multi-scale cross-branch distillation	67.68	78.90	85.62	79.05	88.10	88.20
+ UAV-aware density and hard-region priors	68.15	79.35	85.90	79.52	88.42	88.55
+ Conflict-suppressed reliability routing	68.54	79.72	86.15	80.05	88.68	88.80
+ Consistency and confidence gates	68.92	80.08	86.40	80.40	88.82	88.98
MEKD-UAVSeg (Full)	**69.17**	**80.35**	**86.62**	**80.68**	**88.95**	**89.12**

**Table 7:** Ablation on expert configurations. All variants use the same CNN student and training setting. For dual-expert variants, the fusion and distillation design is kept identical, and only the expert architecture is changed. **Bold** values indicate the best results.

Expert Configuration	UAVid			UDD6		
	mIoU	mF1	OA	mIoU	mF1	OA
Student only	66.45	77.82	84.90	77.50	87.10	87.20
Single Transformer expert	66.98	78.30	85.25	78.10	87.50	87.60
Single Mamba expert	66.85	78.15	85.10	78.25	87.62	87.70
Dual Transformer experts	67.45	78.75	85.55	78.60	87.95	87.95
Dual Mamba experts	67.30	78.60	85.40	78.75	88.05	88.10
Transformer + Mamba experts	**67.82**	**79.05**	**85.80**	**79.15**	**88.35**	**88.40**

**Table 8:** Comparison of different spatial expert designs. All variants use the same Transformer semantic expert, the same CNN student, and the same distillation setting. Only the spatial expert branch is changed, and all expert branches are removed during inference. **Bold** values indicate the best results.

Spatial Expert Design	UAVid		UDD6	
	mIoU	mF1	mIoU	mF1
Dilated CNN expert	68.86	80.05	80.36	88.73
Lightweight attention expert	68.97	80.14	80.45	88.80
Mamba spatial expert	**69.17**	**80.35**	**80.68**	**88.95**

**Table 9:** Ablation study of different training protocols. All variants use the same model components and the same STDC2-FPN student for inference. **Bold** values indicate the best results.

Training Protocol	Warm-Up	Expert KD	Frozen Source	UAVid			UDD6		
				mIoU	mF1	OA	mIoU	mF1	OA
Student only	×	×	×	66.45	77.82	84.90	77.50	87.10	87.20
Direct KD	×	✓	×	67.52	78.85	85.70	79.20	88.40	88.45
Warm-up only	✓	×	×	66.85	78.15	85.15	78.10	87.55	87.65
Warm-up + online KD	✓	✓	×	68.45	79.60	86.15	80.05	88.75	88.85
Proposed two-stage KD	✓	✓	✓	**69.17**	**80.35**	**86.62**	**80.68**	**88.95**	**89.12**

**Table 10:** Feature-stability analysis of different training protocols. Cosine similarity is computed between teacher-side and student features at the last high-level feature stage during the distillation stage. Higher mean similarity and lower fluctuation indicate more stable teacher–student feature alignment. **Bold** values indicate the best results.

Training Protocol	Feature Similarity ↑	Similarity Std. ↓	UAVid mIoU ↑
Warm-up + online KD	0.742	0.031	68.45
Proposed two-stage KD	**0.816**	**0.018**	**69.17**

**Table 11:** Sensitivity analysis of the uncertainty-mask activation epoch $e_{\mathrm{act}}$. The uncertainty mask is disabled before $e_{\mathrm{act}}$ and activated afterwards. **Bold** values indicate the best results.

$e_{\mathrm{act}}$	UAVid		UDD6	
	mIoU	mF1	mIoU	mF1
0	68.79	79.98	80.21	88.70
5	69.03	80.23	80.52	88.86
10	**69.17**	**80.35**	**80.68**	**88.95**
15	69.05	80.22	80.55	88.84
20	68.91	80.08	80.38	88.76

**Table 12:** Sensitivity analysis of the consistency and confidence gate temperatures. **Bold** values indicate the best results.

$\tau_{c}$	$\tau_{r}$	UAVid		UDD6	
		mIoU	mF1	mIoU	mF1
0.1	0.1	68.92	80.10	80.41	88.76
0.2	0.2	**69.17**	**80.35**	**80.68**	**88.95**
0.3	0.3	69.00	80.17	80.46	88.82
0.2	0.1	69.05	80.22	80.53	88.85
0.1	0.2	69.08	80.25	80.57	88.87

**Table 13:** Sensitivity analysis of the Dice loss weight $\lambda_{\mathrm{dice}}$. **Bold** values indicate the best results.

$\lambda_{\mathrm{dice}}$	UAVid		UDD6	
	mIoU	mF1	mIoU	mF1
0.25	69.02	80.18	80.49	88.82
0.50	**69.17**	**80.35**	**80.68**	**88.95**
1.00	68.96	80.12	80.43	88.78

## Data Availability

The datasets used in this study are publicly available. The UAVid dataset and the UDD6 dataset can be obtained from their official repositories. No new dataset was generated in this study. The source code, configuration files, preprocessing details, dataset preparation and split instructions, training and evaluation scripts, random seed settings, checkpoint release notes, and reproduction guidelines for the proposed framework are publicly available at https://github.com/OuYangFengOyf/MEKD.
